# Milestones and turning points in the experience of physical activity throughout cancer care: a qualitative study to inform physical activity promotion

**DOI:** 10.1007/s00520-023-08093-8

**Published:** 2023-11-09

**Authors:** Jany St-Cyr, Kadia Saint-Onge, Isabelle Doré, Lise Gauvin

**Affiliations:** 1 Department of Psychology, Université du Québec à Montréal, Montreal, QC Canada; 2 Social and Preventive Medicine Department, School of Public Health, Université de Montréal, Montreal, QC Canada; 3 Centre de Recherche du Centre Hospitalier de l’Université de Montréal (CR CHUM), Montreal, QC Canada; 4 School of Kinesiology and Physical Activity Sciences, Faculty of Medicine, Université de Montréal, Montreal, QC Canada

**Keywords:** Physical activity, Cancer care, Supportive care, Lived experience, Qualitative research

## Abstract

**Purpose:**

Physical activity (PA) is an important supportive care strategy to manage cancer and treatment-related side effects, yet PA participation is low among people diagnosed with cancer. This study examined patients’, health professionals’, and managers’ perspectives on PA throughout cancer care to glean implications for PA promotion.

**Methods:**

Random selection and purposeful sampling methods allowed for the recruitment of 21 patients (76.2% women) and 20 health professionals and managers (80% women) who participated in individual semi-structured interviews. Interview questions explored facilitators and barriers to PA participation and promotion across the cancer care continuum. Interviews were audio-recorded and transcribed. Then, qualitative thematic analysis was performed.

**Results:**

The analysis produced five main themes describing milestones in PA participation throughout cancer care: (1) Getting Started, (2) Discovering PA Resources, (3) Taking Action, (4) Striving for Change, and (5) Returning to a “New Normal.” The sub-themes underscored turning points, i.e., tasks and challenges to PA participation that had to be overcome at each milestone. Achieving milestones and successfully navigating turning points were dependent on clinical, social, and community factors.

**Conclusion:**

Cancer patients appear to progress through a series of milestones in adopting and maintaining PA throughout cancer care. Intervention strategies aimed at promoting PA could test whether support in navigating turning points could lead to greater PA participation. These findings require replication and extension, specifically among patients who are men, younger adults, and culturally diverse.

**Supplementary Information:**

The online version contains supplementary material available at 10.1007/s00520-023-08093-8.

## Introduction

Physical activity (PA) has been shown to be safe and beneficial for both the physical and mental health of people diagnosed with cancer [1–3]. PA represents an effective component of supportive care for cancer patients before [4], during, and after treatments [5, 6]. Engaging in PA reduces patients’ risk of cancer recurrence, optimizes rehabilitation, and improves physical fitness, quality of life, and survival [3, 5, 6].

Despite benefits and evidence-based guidelines (see Table 1 [7]), few cancer patients cumulate the recommended levels of PA [8–12]. In addition, current practice does not systematically address patients’ needs that go beyond medical treatment [13]. Yet, health professionals can play a major role in PA engagement throughout cancer treatment and survivorship [14, 15] especially oncologists [16]. Frequently reported barriers to promoting PA include lack of time, unclear referral pathways, low perceived patient interest, and little knowledge of oncology PA guidelines and services within and outside clinical settings [17–19].

**Table 1:** Physical activity guidelines for people with chronic conditions according to the American College of Sports Medicine [7]

Guidelines
• Avoiding daily inactivity
• Engaging in at least 90 min (3 × 30 min) of moderate-intensity aerobic PA per week
• Engaging in at least 2 weekly sessions of muscle-strengthening activities involving all major muscle groups

An overarching health promotion strategy to increase PA participation among cancer patients requires an integrated understanding of the facilitators and barriers to PA in real cancer care settings as well as in the community [20]. PA programs designed for cancer patients represent important opportunities for engaging in PA. When led by exercise specialists, they can help fill the service gap by providing expert knowledge, personalized recommendations, and motivational support [21]. However, PA programs are sparsely offered in oncology settings [22, 23], few health professionals promote and discuss PA with patients [17], and little is known about PA support in the community.

PA promotion for people diagnosed with cancer must be strengthened in clinical care settings and bolstered with a community-setting approach. Previous work investigating cancer patients’ perspectives of PA program acceptability, appreciation, and efficacy consistently shows that specialized staff, social support, peer support, and a person-centered approach are key to compliance and adherence to such programs [23–26]. Tailoring them to individual needs is key to success and improves PA outcomes [8, 23, 27]. The end of active treatment for cancer patients can be challenging for maintaining PA participation and is marked by concerns about losing support from hospital staff and feelings of isolation and vulnerability [28–30]. For those who were working or returning to work, cancer and its treatment may have affected their ability to perform work-related tasks and thus cause distress [31]. As a result, strategies to promote PA inside and outside clinical settings must capitalize on facilitators and overcome barriers experienced by patients and health professionals throughout the sequence of diagnosis, treatment, and survival. These challenges are best understood synergistically, in line with the patient-as-partner approach in health care [32].

Hence, there are several gaps in the current literature on supporting and promoting PA among people diagnosed with cancer. First, few studies have examined oncology practitioners’ perspectives on how PA is addressed at different points throughout the cancer care process [20]. Garnering their perspectives could shed new light on facilitators and barriers to PA promotion faced or witnessed in their practice [21]. Second, studies including health professionals’ experience in promoting PA tend to consider cancer patients’ experience of PA programs independently of health professionals’ experience [20]. The successful design and implementation of services depend on joint consideration of these perspectives to ensure that services are beneficial, widely accessible, promoted, and adhered to [33, 34]. Third, little is known regarding the perspectives of hospital managers. These managers are well-positioned to provide insights into organizational policies, procedures, climates, and regulations to implement PA services or referrals inside and outside clinical settings [35]. Combining these perspectives could provide a richer, more holistic understanding of PA promotion for people diagnosed with cancer. We therefore sought to examine patients’ as well as health professionals’ and managers’ complementary perspectives on PA experience throughout cancer care.

## Methods

### Research design and setting

We conducted a qualitative cross-sectional study [36]. This design allowed us to garner an in-depth understanding of PA through cancer care from the perspectives of cancer patients’ as well as those of health professionals and managers involved in cancer care delivery or service organization [19, 35, 37]. This study was part of a larger project examining PA and cancer care which is described in detail elsewhere [38].

### Participants

We solicited two convenience samples to recruit patients as well as health professionals and managers [36]. To ensure we garnered perspectives of patients with experience of PA through cancer, we collaborated with *Fondation Virage*. This non-profit foundation offers various services for cancer patients at the *Centre Hospitalier de l’Université de Montréal* (CHUM) including in*-treatment PA programs, rehabilitation PA programs,* and *maintenance PA programs*. Exercise specialists supervise personalized sessions according to the patients’ stage of care, while monitoring their overall health condition. The breadth and varied focus of programs offered in this center provided an appropriate setting for recruiting patients with a wide range of PA experiences.

Potential participants were required to be aged 18 years or over. Patients had to be diagnosed with cancer and enrolled in or have completed a *Fondation Virage* PA program. We aimed to recruit 20 participants from a random selection of 60 patients (30 men and 30 women) having participated in the *Fondation Virage* PA program between 2017 and 2019. *Fondation Virage* staff and research team members approached potential participants to enquire about their interest in participating in a one-on-one research interview. Potential health professionals and managers were identified from a pool of experts known to the research team and the *Fondation Virage* staff. We sought health professionals and managers having previous experience with oncology care or PA service delivery in a hospital setting. All listed individuals were invited to participate in interviews.

### Data collection

Three interviewers were trained in conducting qualitative interviews. Interviewers were not involved in the delivery or administration of PA programs and were not acquainted with participants. Between June 2019 and October 2020, they conducted individual, semi-structured interviews that lasted an average of 35 min (MD = 35.0, SD = 10.5, Min = 17.0, Max = 57.0). Interviews were conducted in person until the onset of the COVID-19 pandemic and then by telephone or teleconference using Zoom Meetings software. Participants responded to a series of questions about PA and cancer. In this investigation, we focused on barriers and facilitators to PA participation throughout cancer care (including the PA program) and on health professionals’ role in supporting and promoting PA. Health professionals and managers were asked about PA promotion and delivery among people diagnosed with cancer and about how patients described their PA participation and its promotion (see [38] for the full study’s interview guides). Interviews were audio-recorded and transcribed verbatim by an external service provider.

### Data analysis

Data were analyzed by four research assistants using a hybrid thematic analysis approach [39] with Dedoose qualitative analysis software and Microsoft Excel. Analyses aimed at identifying common themes and sub-themes among patients and health professionals and managers. To uphold qualitative rigor, research assistants kept and shared analysis journals, attended weekly interrater meetings where they discussed produced codes and themes, and confirmed or readjusted emerging interpretations until consensus was met [40]. We used a hybrid approach for the first round of coding [36]. Deductive coding served to identify meaningful text excerpts using an evidence-based guide: the Tailored Implementation for Chronic Diseases checklist (TICD [41]). The TICD checklist was used to facilitate initial coding as it provides a systemic perspective of the essential barriers and facilitators to consider for program implementation including promotion. Simultaneously, inductive coding was applied to identify novel codes and latent themes that characterized patients’, health professionals’, and managers’ experiences with PA. Patients’ as well as health professionals’ and managers’ perspectives were analyzed simultaneously to produce a global portrait. Coders observed an initial pattern of key moments in participants’ experience of PA through cancer. First, coders identified subthemes representing *turning points* or critical moments for change. Second, turning points were grouped into chronological themes representing *milestones* in the PA experience through cancer care. The main author then reviewed the data twice to identify perceived barriers and facilitators for each milestone.

### Ethics approval

The CHUM’s research ethics board reviewed and approved this study protocol (CER# 17.238). Participants provided written informed consent for audio-recorded interviews.

## Results

Of the 41 participants, 21 were patients (cancer sites: breast, prostate, thyroid, lungs, and other; women = 16 (76.2%)), and 20 were health professionals or managers (women = 16 (80%)) working in oncology, nursing, cardiology, specialized exercise delivery, public health, and management. The analysis produced five milestones and eleven turning points in the experience of PA through cancer care. Table 2 outlines the milestones and presents illustrative quotes for each turning point.

**Table 2:** Participant quotes illustrating milestones and turning points of the experience of physical activity through cancer care

Milestone	Turning point	Participant type	
		Patients	Health professionals and managers
Getting Started	Laying the foundations	“At 25 years old, when I’d feel down, I went for a run. I wasn’t a big maniac, but I had already experienced that it helps me a lot. […] I was in a bad mood. I’d tell my boyfriend off. I knew I wasn’t nice. I told myself: ‘Ah, wait a minute, I’m going to run for half an hour and feel better when I come back.’ I already knew the benefits of exercise, both physical and mental.” Patient 1	“There’s several sporty people on my team. Half [of them] jog, some do marathons. So, for sure it’s people who will encourage [the patients to do PA]. How well equipped are they? For that, maybe that’s weaker. […] Some people are very, very sold on the idea, like some of my colleagues. But again, it also depends on the patient that I will be dealing with. I would tell you that most of the members of my department are quite positive about PA.” Health professional or manager 1
	Taking care of myself/themselves, at what cost?	“I wasn’t sure what to expect. The first time I was in it, honestly, I didn’t know. I was worried and especially: Is it really worth doing this? I had no energy. And when you don’t have energy, you have even less. You say to yourself: ‘I wouldn’t have the energy to do this either.’ So, it was kind of convincing myself that it’s going to be positive.” Patient 2	“Often, [patients] are afraid that they will be more tired. Because fatigue is the most common symptom. Energy isn’t restored. So, it’s a little paradoxical to ask them to do PA. Makes them a little afraid of not being able, of being more tired. So, we reassure them a lot about the dosage. In fact, they come to see us to learn how to better balance not only the activities they are going to do with us but also all the activities they do in their daily lives.” Health professional or manager 2
Discovering PA Resources	Mustering up resources	“When I was diagnosed, I did some research. I first chose the hospital where I wanted to have all the tests because it was close to my work. I had also called other places. And then, it was, yes it was a pamphlet at the hospital that I learned about [the program].” Patient 1	“We need a professional, such as a kinesiologist or physiotherapist, who is there with us to equip us. At the same time, professionals all have this responsibility to [address PA with patients]. I mean, it’s not for one person to do this. I think we all have this responsibility, but I have colleagues who will say: ‘Yeah, but I’m overloaded, I’m struggling’. That’s more the issue. But […] our role is not only to assess and intervene, it’s also to promote and prevent.” Health professional or manager 3
	Finding the right time	“Honestly, I would say that before you have treatments, you’re pretty stressed and you don’t know what’s going to happen. There’s a lot of worry, a lot of fears, you’re not in the right headspace to jump into a PA program […] because you’re not there. You’re in combat.” Patient 2	“When the patient receives their diagnosis, in the beginning, we can’t center them on structured activities because, […] as soon as you’re told that, you see the end. So, at that moment, the patient needs more to rebuild themself mentally. […] We have to leave some time for them to absorb the diagnosis and climb the hill” Health professional or manager 4
Taking Action	Being supported to better evolve	“I certainly felt heard, supported, and supervised. Then, I really liked the fact that we were offered exercises that were at the level of what I could do at that time. They told me: ‘There are people who have gone through what you’re going through and look what they are doing now, well look at what you can do’. You really have to accept your physical condition for what it is today and that it won’t necessarily stay that way. But I have to give myself time and I have to accept that my physical condition is no longer what it was before. Then, they were introducing me to exercises that were really within my reach after all. And to be able to have the opportunity to see myself evolve in the right direction.” Patient 3	“What I find very interesting is that our patients who go to exercise with a kinesiologist have a program there that is adapted to their situation. So to feel that it’s adapted to them, and to have goals that are realistic. Because there is everything ‘how I was before having my cancer when I was really to my liking or, in any case, more to my liking than today’ versus ‘I am very limited because of my cancer’. So, you go from there and the patient has their personal markers with their goals which makes them see their improvements.” Health professional or manager 5
	Regaining control	“It helps you feel alive in your body because, at that point, you’re not sure if you’re going to live. It was for me a way to really feel the life in my body. It’s really major. Then, I felt the physical and psychological benefits. Like I told you, to feel life, even to feel that you have muscle tone. And the psychological side of being like: ‘Okay, I’m not completely screwed. I still exist. I can still move. I can still do something.’ There were all these aspects of exercising that motivated me.” Patient 4	“They’re going to say, ‘I’m going to take charge. I quit smoking, I do this, and I do that. I start training to do that’. For them, this is extremely important because they don’t feel like they’re doing nothing. That’s the whole idea of the patient partner: it’s that they will be partners. I can’t exercise for them. I will operate them, that’s for sure. But the exercise, they have to do it [themselves]. (…) And after that, the physical effect will also be important on morale. But, doing something, not staying in, ‘I’m waiting, I’m taking the exams, I’m going to do etc.’ The fact of no longer suffering and of being an actor… Even for small things, essential things.” Health professional or manager 6
	Feeling understood and accepted	“But there’s something that’s not negligible too, it’s the people. The other people we meet and who have similar backgrounds. I found that it was worth it for me, to go [to the hospital] rather than being mixed in a group of people who don’t necessarily have the same background […]. There’s also the part there too that was supportive, of meeting people who do the same thing, who experience the same thing as us or similar. […] We encouraged each other because sometimes there’s one that wasn’t doing so well and all that. We called and encouraged each other, we knew, we waited for each other in training.” Patient 5	“There are a lot of people who bring up the importance of being able to do this in a group too. They can interact with other participants. They find it hard sometimes, they don’t want to talk about it with their loved ones, they don’t necessarily want to put that weight on them, that negative pressure. Then, support groups for example, are not always something that interests patients. On the other hand, being able to be in a group with other people who are going through the same situation, to share a little but in good spirits, in a positive atmosphere. ‘We are here to do each other good’; I think it’s great. [Patients] often tell us about it.” Health professional or manager 7
Striving for Change	Taking the time to prepare	“In fact, if the program could last longer. If we could be entitled to it in other stages Like longer. I understand it’s already good, it’s good. But, when that ends, when we leave you, there are other stages that are related to our limitations, and we can still have specific needs.” Patient 4	“We see the patient three times a week. We create a really strong bond with them and with all the patients. Also, there is a synergy that is created. They’re all in the same medical condition, the same health condition, the same age group, roughly. So that there are links that are created. And that maintains the patient’s motivation. We look like clowns the way we try to keep our patients motivated. But the problem with that is, they don’t want to go after that anymore. We can’t train them forever. We keep them for maximum one year. After that, you have to help them stand on their own feet and find resources. Sometimes, at the end of the line, they just don’t want to go.” Health professional or manager 8
	Finding resources that meet my needs	“It’s easier when you have a structure, a schedule, something to respect, an appointment. […] With the pandemic, everything stopped and you had to motivate yourself. At first, I was successful because I had an exercise session to do every day, […] but to do a little more, well, you had to motivate yourself [more]. At first, going for a walk with my husband helped […] but after that, the children’s school started. Again, it was a free-for-all: there was no schedule anymore. I was completely exhausted. All summer long, it was difficult to go back [to my PA habits]. But I hope that with the start of the school year, I’ll be able to organize myself.” Patient 6	“There are people who said that above all they wanted to do it in a safe environment. Being safe for them is in the hospital or with a kinesiologist. They feel more secure. When I think about it, I think it’s a partnership between the hospital environment and the community that’s needed. I think we have to make connections. I say that, but I haven’t validated it with everyone. But I think we have to make links with certain external organizations.” Health professional or manager 9
Returning to a “New Normal”	Applying what I have learned	“I’ve learned the importance of long-term benefits. It decreases negative thinking, we’re fitter, we have more endurance. However, after the program, I was less used to exercising. The heat of summer and being home alone, I quit. […] You don’t want to put in efforts and your willpower weakens, but when exercise is all you have to feel better, motivation becomes easier.” Patient 7	“Patients will be extremely sensitive to the quality of life, sensitive to its importance. What is important, what isn’t. And if they’ve gained that knowledge, during those three to six months [of PA] and that it has helped them, it will happen on its own because, don’t forget, they’ll live with [cancer] their whole life. It’s not because we’re going to follow them for a year, two years, five years, or ten years. They’ll have this with them all their life. So, it’s really to realize that [AP] improves their quality of life. […] Anything that improves the quality of life, they will take it.” Health professional or manager 6
	Managing external expectations	“Afterwards, I signed up for [another PA] program that the Foundation offers […]. But that, I didn’t really like my attitude there because I was back at work. My job is […] very demanding. When I restarted, the doctor told me: ‘You will go [to work] gradually.’ I couldn’t do it gradually. A week later I was full time and very tired. Then, people are selfish, and I wasn’t able to tell people: ‘I have an activity, I have to leave at such and such a time.’ And said: ‘No.’ It was like that all the time, all the time.” Patient 8	“It’s fine as long as they don’t go back to work. Worse when they go back to their work routine, that’s where we lose them. […] It’s not always easy to keep an active life through all of this. If on top of that they have to return to their past life, this whirlwind, it’s an important issue. I think we should maybe approach the employers and do a little more education, say: ‘Okay, the person is coming back to work. You need to give them a chance, give them a moment to learn from and stay active both at work and in their daily life.’” Health professional or manager 2

Free translation from the research team

### Milestone 1: Getting Started

This starting point in patients’ PA trajectory through cancer care appears to be significantly influenced by the degree of positiveness of their previous and present experiences of PA, more so than any other milestone. Health professionals and managers described factors that influence whether a discussion about PA was initiated. The following turning points describe how experiences led participants to start considering PA as a self-care strategy.

#### Turning point: laying the foundations

This turning point describes both past and present factors that influence engagement in the PA program. Patients noted that their respective types of cancer, treatments, and side effects affected PA participation differently. Some of them reported having to decrease the intensity of PA due to fatigue or pause it due to the treatments they were receiving. Knowledge about PA and one’s relationship with it before being diagnosed with cancer also influenced enrolling in the PA program. Most patients mentioned that they were already physically active before their cancer diagnosis. Thus, they were motivated to maintain their physical fitness, improve their well-being, and give themselves the best chance to beat cancer. For example, a patient explained that, in the past, they used PA as a coping strategy to clear their mind when they were angry. Thus, they were motivated to engage in PA to improve their well-being during cancer care. Health professionals and managers stated that their ability and willingness to promote PA to their patients depended heavily on their knowledge of PA benefits and their own experience of PA, their level of familiarity with each patient, and their knowledge of available PA services.

#### Turning point: taking care of myself/themselves, at what cost?

This turning point represents the questions, doubts, and expectations that patients experienced regarding the practice of PA during treatment. Knowledge about PA and its benefits was reported to be motivating. Yet, fears and concerns about its potential effects were common. Fear of injury or overtraining could thwart willingness to engage in PA. Health professionals and managers were aware of these concerns. In fact, some shared the same concerns. Health professionals and managers who expressed strong beliefs in the value of PA indicated that they had to adapt their patient counseling practices by acknowledging, counseling, and educating patients to reduce these fears.

### Milestone 2: Discovering PA Resources

This theme describes participants’ reports of a stage in the trajectory where they needed to pool resources to access the PA program given potential difficulties along the way. Specifically, time, energy, willingness, and opportunity were mentioned as needed resources.

#### Turning point: mustering up resources

This turning point describes the means currently in place to promote PA to patients and their effects. Patients mentioned various ways in which they tracked down the PA program. They learned about the program namely through relatives, pamphlets, information groups, and online searches. Several patients stated that none of their physicians addressed PA with them or mentioned the hospital-based program. Thus, they conducted their own search to get information about being physically active during cancer treatments. They stated that searching for information about PA requires much willpower and resources such as time and energy. On the other hand, health professionals and managers said that they lacked specific tools such as a leaflet, a directory of available PA programs and resources, or a reminder to promote PA to patients. Like patients, some felt that information about PA participation during cancer treatment was not widely available. Overall, health professionals and managers said that information about PA was not promoted to all cancer patients because PA promotion procedures (e.g., referral process) and their own role in the process were not clearly defined.

#### Turning point: finding the right time

This turning point describes overcoming the shock of receiving a cancer diagnosis, its side effects, and its impact on PA participation. Patients stated that in the “*storm*” triggered by a cancer diagnosis, emotions, stress, and worries took over. It was hard for them to find a good time to think and talk about PA. Similarly, health professionals and managers said that it can be difficult for them to find the appropriate time to talk about PA. Some found it difficult at times to know when patients are emotionally ready to address PA. Health professionals and managers also described being overloaded by other tasks and did not think about or have time to discuss PA with patients.

### Milestone 3: Taking Action

This milestone describes patients’ experience during the PA program. At this stage, patients take action on their health, but different factors can influence their participation such as feeling supported and empowered. Given the personal nature of this milestone, it was mainly informed by patients’ perspectives.

#### Turning point: being supported to better evolve

This turning point describes participants’ perceptions of how exercise specialists affect patients’ progress in the PA program and in their lives. Patients who were both physically active and inactive before diagnosis appreciated adapted and personalized exercises. A nurturing relationship had developed between kinesiologists and patients which made patients feel heard and safe. They mentioned that the hospital environment, the personalized support of expert kinesiologists trained in oncology, and the follow-ups adapted to their needs and their health conditions allowed them to feel confident, supported, reenergized, and safe. In turn, health professionals and managers were aware of the importance of tailoring the program to each patient’s situation and the benefits that it has for them. They were confident in the quality and safety of services that exercise specialists trained in oncology could provide.

#### Turning point: regaining control

This turning point describes experiences and observations of empowerment that were perceived to come from participating in the program and in PA more generally. Patients mentioned that setting goals and perceiving physical and psychological benefits allowed them to exercise control over their situation and to gain an even more positive perceptions of PA. This sense of control was also fostered by the acquisition of new knowledge about PA and by their increasing awareness of a new reality: the reality of surviving cancer. Regaining control over their bodies and their health allowed them to “*weather the storm*.” Health professionals and managers also mentioned that PA practice and its benefits motivated patients throughout treatment. This new-found motivation, they said, played a considerable role in recovery since patients must be “*active actors*” to improve their health.

#### Turning point: feeling understood and accepted

This turning point describes the importance of social support for patients in their PA experience through the cancer treatment and survival process. Participants described how support from family and friends rested on a delicate balance between tailoring support to their needs and overwhelming them with advice. The most important source of support mentioned by patients was from other people diagnosed with cancer participating in the PA program. The presence of others going through a similar situation was a bonding experience as well as a great source of motivation. Health professionals and managers recognized the important role social support played in the PA group program. They said that it can be easier for patients to talk about their experience with people who are also going through it, in a friendly space where they can feel “*a sense of normalcy*.”

### Milestone 4: Striving for Change

This milestone describes a critical point at which the program comes to an end. It is a time of great change as patients prepare to transition from PA in a hospital setting to PA in the community and during which patients need even greater autonomy. In this milestone, the role of health professionals and managers becomes more distal, but their presence is still required to facilitate the transition.

#### Turning point: taking the time to prepare

Preparing for the transition into the community is a key, but time-consuming, moment to ensure that PA participation continues after the program is over. Patients stated that they were not sufficiently prepared to transition away from the program and would like for it to last longer. Patients and health professionals and managers agreed on the importance of preparing for the transition. Some health professionals and managers believed that it is necessary to support patients during the transition but to gradually give them more autonomy, while others believed that the remaining work is more in the hands of the patients, who must “*cut the cord*.”

#### Turning point: finding resources that meet my needs

This turning point describes a time when patients sought out resources that met their needs to maintain their PA practices. To do so, participants mentioned needing resources of all types to increase their motivation and facilitate PA. These resources varied depending on each patient’s situation: money, time, social support, self-management competency, and safe and adapted services in the community. Health professionals and managers felt that it was difficult to find suitable external resources that met the needs of cancer patients on top of their own professional standards. Some mentioned that a bridge must be built between the hospital setting and the community so that patients can continue to do PA.

### Milestone 5: Returning to a “New Normal”

This last milestone describes the critical point when patients attempt to return to their lives after the program and treatment have ended. Because surviving cancer changes a person both physically and mentally, participants said, the “*normal*” life they had before their diagnosis is now transformed into a “*new normal*.” Health professionals and managers shared their perspectives on factors influencing patients’ maintenance of PA following the program.

#### Turning point: applying what I have learned

This turning point describes how patients attempted to apply what they had learned in the program to their daily routine. Patients said that they learned new exercises and how to adapt them to their needs. They also gained and reinforced their knowledge about the benefits of PA and its importance to health and well-being. Despite this knowledge, they reported that it can be difficult to stay motivated. Among the factors that hindered their motivation, they named: feeling alone, managing their time, getting through the COVID-19 pandemic, and weathering harsh climate (e.g., ice and snow during the winter). Health professionals and managers believed that patients learned new exercises and how to adapt PA to their needs, and, most importantly, that they understood the importance of having healthy lifestyle habits. Even so, they were aware that it can be difficult for patients to put what they have learned into practice while readjusting to their daily lives.

#### Turning point: managing external expectations

This turning point depicts the patients’ attempts to resume their daily activities while meeting external expectations. Several patients mentioned that during the PA program, they learned to accept the physiological and psychological changes caused by cancer and its treatments. Patients also reported that their abilities and skills had changed after treatment, but that those around them expected them to do the same things they did before their cancer diagnosis. For example, some said that their boss expected them to do as much work as before or even more to make up for missed work. Some also mentioned that they could not stand the external expectations and pressure, so they chose to let go of negative people, change jobs, or focus more on their well-being. Health professionals and managers emphasized how patients’ social environment post-treatment presents barriers to maintaining PA. They added that there was a lot of work to be done with employers to enforce a gradual return to work and to educate them about cancer survival.

 We observed that the transition from one milestone to another was not unidirectional or linear. Rather, there appeared to be an overlap between some milestones and turning points, and it was possible to go back and forth between them. This was also the case for the barriers and facilitators identified for each milestone, which are listed in Table 3.

**Table 3:** Facilitators and barriers at each milestone in the physical activity experience throughout cancer care

Milestone	Facilitators	Barriers
Getting Started	- Knowledge about PA and its benefits - Positive experience of PA - Patients’ motivation to engage in PA - Good patient-health professionals’ relationship ◦ Acknowledgment of patient’s fears ◦ Communication (language clarity, listening, etc.) ◦ Education about PA in cancer	- Doubt that PA will help ◦ Fear of injury - Lack of knowledge of available PA services - Cancer and treatment-related side effects
Discovering PA Resources	- Volition to search for resources - Interest in PA	- Unequal access to PA resources ◦ Lack of specific PA promotion tools (e.g., leaflet, directory of PA resources and programs) ◦ Not clearly defined procedures for promoting PA - Cancer diagnosis “storm” ◦ Lack of time and energy ◦ Difficulty finding the right time to talk about PA - Overburdened health professionals
Taking Action	- Patients centered approach ◦ Professional support ◦ Social support - Secure environment ◦ Supervision by kinesiologists trained in oncology ◦ Adapted and personalized exercises - Tangible PA benefits	- Family and friends overwhelming patients with advice
Striving for Change	- At home PA tools (e.g., list of exercises and plan)	- Lack of preparation to transition from hospital to community settings - Lack of external resources adapted to cancer patients needs
Returning to a “New Normal”	- Patients’ acquired knowledge ◦ Practical knowledge (i.e., exercises and how to adapt them to their needs) ◦ Theoretical knowledge (i.e., benefits of PA for physical and psychological health) - Patients’ acceptance and better awareness of their body and health - Patients redefining their life priorities	- Difficulty for patients to stay motivated to do PA while resuming their daily activities ◦ Loneliness ◦ Difficulty in managing time ◦ Contextual factors (e.g., weather conditions, Covid-19 pandemic) - Family and friends’ expectations for recovery - Workplace expectations

## Discussion

We examined patients’ as well as health professionals’ and managers’ combined perspectives on PA participation and promotion throughout cancer care. Using a hybrid thematic analysis approach of interview data, we identified five milestones characterized by unique turning points which describe patients’ experience of PA throughout the sequence of diagnosis, treatment, and survivorship. Below, we discuss each milestone in relation to previous work.

In Getting Started, our data align with previous work showing that patient-reported barriers to PA include a lack of knowledge about how to exercise, a lack of awareness of its benefits, and uncertainty about the evidence for its benefits [19, 28]. Many patients in our study reported having fears about the safety of PA, highlighting a gap in information dissemination concerning PA in cancer. Demystifying these doubts and fears is essential because they can be a barrier to engaging in PA and maintaining PA practice during the cancer journey [28]. As suggested by Santa Mina and colleagues [42], developing varied promotional and educational resources about PA in cancer could effectively contribute to reaching patients, especially those who are inactive and have limited knowledge about PA.

For many patients in our study, Discovering PA Resources required volition and a very proactive approach on their part. Similarly to other studies [17–19], our findings show that the referral process to PA programs was inconsistent. In addition, health professionals and managers had concerns about the right time to discuss PA with patients given the emotional burden of a cancer diagnosis. To overcome these barriers, Santa Mina and colleagues [42] suggested establishing a pathway that encourages interprofessional communication and collaboration between health and cancer-trained PA professionals to provide safe and effective resources. Considering our results, we suggest implementing an advocacy strategy in which cancer survivors with lived experience of cancer care and PA services facilitate PA promotion along with patients, health professionals, and managers. Their experiential knowledge could help tailor promotion efforts to the lived experience of cancer care and PA (e.g., peer mentoring; for a meta-analysis see [43]).

While Taking Action during the program, the presence of kinesiologists specialized in oncology made patients feel safe and confident to exercise as found by Dennett and colleagues [25]. The customization of the program to meet patients’ needs also contributed to these feelings. Moreover, as found in other studies [23, 26], the presence of other patients created a feeling of belonging and was an important source of motivation. Also in line with previous research, we found that throughout the PA program, patients developed an even more positive perception of PA [24, 44–48]. Indeed, their perceived physiological and psychological benefits gained during the program contributed to lessening doubts about PA, thus removing an important barrier to participation.

In Striving for Change, our results highlight the importance of gradually preparing for the transition from the hospital to the community. The development of adapted and safe oncology PA services in the community could facilitate the transition, allowing patients to develop new relationships outside the hospital setting. For example, implementing a structured transition process to community-based exercises with transferable tools, peer support, and ongoing monitoring could ease the transition [25, 30]. Schmidt and colleagues [30] suggested structuring the transition process by also providing supervised exercise sessions at a community fitness center, and strategies to facilitate group sustainability such as a buddy system.

Returning to a “New Normal” was very challenging for several patients. They stated that learning to accept themselves has great value for their psychological health, which was also documented elsewhere [49]. Together, these data suggest that preparing to reintegrate one’s routine should include aspects such as expectations for what’s to come as well as ways to circumvent anticipated barriers. On the other hand, when returning to “normal,” patients were confronted with the demands of the outside world. The work environment was a particularly challenging aspect. Some cancer patients and survivors return to work prematurely due to pressure from their employer or insurance company, overwork due to financial anxiety, or neglect other aspects of their lives because their abilities, priorities, and interests have changed [50]. Some authors have suggested that employers implement accommodations (e.g., modifying work schedules or tasks) to facilitate functioning at work [31, 51]. However, the stigma and discrimination cancer patients and survivors experience at work could deter the offer and acceptance of such accommodations [50]. Thus, we suggest that PA in cancer care promotion should also aim to reduce cancer stigma by educating the general population about life after cancer and the impacts of performance-heavy social norms on physical and psychological health. Employers could contribute to this by offering PA opportunities that are inclusive to people diagnosed with cancer.

## Clinical implications

This study identified critical milestones and turning points in the experience of PA through cancer care that may be better maneuvered by overcoming barriers while leveraging facilitators. These milestones and turning points could serve to select, adapt, or inform the development of PA promotion tools that are suited for use throughout the continuum of cancer care. In keeping with the patient-as-partner approach in health care [32], this study’s results could provide meaningful information to better situate patients’ needs for PA and to tailor promotion according to identified time points. For patients, they could serve as a roadmap of what to expect based on what others have experienced. Intervention strategies aimed at promoting PA could test whether support in navigating turning points could lead to greater PA participation.

## Limitations and future directions

This study has limited generalizability. Garnered perspectives of PA are limited to patients having participated in a supervised hospital-based PA program in the city of Montreal, Canada. Secondly, most patients were already physically active before their cancer diagnosis. Lastly, the sample included more women than men. Future studies should include people diagnosed with cancer who did not participate in a PA program or in PA altogether, and those who were physically active by means other than supervised programs. These findings should be replicated and extended by including participants with diverse individual characteristics such as patients who are men, younger adults, and of various cultural and ethnic identities.

## Conclusion

The integrated combination of patients’ and health professionals’ and managers’ perspectives provided a more comprehensive understanding of PA participation among individuals diagnosed with cancer. This allowed us to uncover critical milestones and turning points throughout the PA in the cancer care continuum. Identified milestones and turning points could serve to meaningfully inform and tailor PA promotion for people diagnosed with cancer.

### Supplementary Information

Below is the link to the electronic supplementary material.

Supplementary file1 (DOCX 183 KB)

## References

[1] Dittus KL, Gramling RE, Ades PA (2017). Exercise interventions for individuals with advanced cancer: a systematic review. Prev Med.

[2] Schmitz KH, Courneya KS, Matthews C (2010). American College of Sports Medicine roundtable on exercise guidelines for cancer survivors. Med Sci Sports Exerc.

[3] Segal R, Zwaal C, Green E (2017). Exercise for people with cancer: a systematic review. Curr Oncol.

[4] Giles C, Cummins S (2019). Prehabilitation before cancer treatment. BMJ.

[5] Campbell KL, Winters-Stone K, Wiskemann J (2019). Exercise guidelines for cancer survivors: consensus statement from International Multidisciplinary Roundtable. Med Sci Sports Exerc.

[6] McTiernan A, Friedenreich CM, Katzmarzyk PT (2019). Physical activity in cancer prevention and survival: a systematic review. Med Sci Sports Exerc.

[7] Patel AV, Friedenreich CM, Moore SC (2019). American College of Sports Medicine roundtable report on physical activity, sedentary behavior, and cancer prevention and control. Med Sci Sports Exerc.

[8] Bullard T, Ji M, An R (2019). A systematic review and meta-analysis of adherence to physical activity interventions among three chronic conditions: cancer, cardiovascular disease, and diabetes. BMC Public Health.

[9] IJsbrandy C, Ottevanger PB, Gerritsen WR (2021). Determinants of adherence to physical cancer rehabilitation guidelines among cancer patients and cancer centers: a cross-sectional observational study. J Cancer Surviv.

[10] Littman AJ, Tang M-T, Rossing MA (2010). Longitudinal study of recreational physical activity in breast cancer survivors. J Cancer Surviv.

[11] Midtgaard J, Baadsgaard MT, Møller T (2009). Self-reported physical activity behaviour; exercise motivation and information among Danish adult cancer patients undergoing chemotherapy. Eur J Oncol Nurs.

[12] Stevinson C, Lydon A, Amir Z (2014). Adherence to physical activity guidelines among cancer support group participants. Eur J Cancer Care (Engl).

[13] Handberg C, Jensen CM, Maribo T (2017). Lack of needs assessment in cancer survivorship care and rehabilitation in hospitals and primary care settings. J Clin Med Res.

[14] Jones LW, Courneya KS (2002). Exercise discussions during cancer treatment consultations. Cancer Pract.

[15] Leach HJ, LeBreton KA, Wurz AJ (2020). Exploring cardiologists’ and oncologists’ exercise recommendation and referral practices. Health Educ J.

[16] Elshahat S, Treanor C, Donnelly M (2021). Factors influencing physical activity participation among people living with or beyond cancer: a systematic scoping review. Int J Behav Nutr Phys Act.

[17] Alderman G, Semple S, Cesnik R, Toohey K (2020). Health care professionals’ knowledge and attitudes toward physical activity in cancer patients: a systematic review. Semin Oncol Nurs.

[18] Hardcastle SJ, Kane R, Chivers P (2018). Knowledge, attitudes, and practice of oncologists and oncology health care providers in promoting physical activity to cancer survivors: an international survey. Support Care Cancer.

[19] Nadler M, Bainbridge D, Tomasone J (2017). Oncology care provider perspectives on exercise promotion in people with cancer: an examination of knowledge, practices, barriers, and facilitators. Support Care Cancer.

[20] Chan RJ, Nekhlyudov L, Duijts SFA (2021). Future research in cancer survivorship. J Cancer Surviv.

[21] Cantwell M, Walsh D, Furlong B (2018). Healthcare professionals’ knowledge and practice of physical activity promotion in cancer care: challenges and solutions. Eur J Cancer Care (Engl).

[22] Canestraro A, Nakhle A, Stack M (2013). Oncology rehabilitation provision and practice patterns across Canada. Physiother Can.

[23] IJsbrandy C, Hermens RPMG, Boerboom LWM (2019). Implementing physical activity programs for patients with cancer in current practice: patients’ experienced barriers and facilitators. J Cancer Surviv.

[24] Avancini A, Tregnago D, Rigatti L (2020). Factors influencing physical activity in cancer patients during oncological treatments: a qualitative study. Integr Cancer Ther.

[25] Dennett AM, Peiris CL, Taylor NF (2019). ‘A good stepping stone to normality’: a qualitative study of cancer survivors’ experiences of an exercise-based rehabilitation program. Support Care Cancer.

[26] McDonough MH, Beselt LJ, Daun JT (2019). The role of social support in physical activity for cancer survivors: a systematic review. Psychooncology.

[27] Ottenbacher AJ, Day RS, Taylor WC (2012). Long-term physical activity outcomes of home-based lifestyle interventions among breast and prostate cancer survivors. Support Care Cancer.

[28] Cantwell M, Walsh D, Furlong B (2020). Physical activity across the cancer journey: experiences and recommendations from people living with and beyond cancer. Phys Ther.

[29] Jefford M, Karahalios E, Pollard A (2008). Survivorship issues following treatment completion—results from focus groups with Australian cancer survivors and health professionals. J Cancer Surviv.

[30] Schmidt MLK, Østergren P, Cormie P (2019). “Kicked out into the real world”: prostate cancer patients’ experiences with transitioning from hospital-based supervised exercise to unsupervised exercise in the community. Support Care Cancer.

[31] Alfano CM, Kent EE, Padgett LS (2017). Making cancer rehabilitation services work for cancer patients: recommendations for research and practice to improve employment outcomes. PM R.

[32] Karazivan P, Dumez V, Flora L (2015). The patient-as-partner approach in health care: a conceptual framework for a necessary transition. Acad Med.

[33] Garratt AM, Solheim E, Danielsen K. (2008) National and cross-national surveys of patient experiences: a structured review. Rapport nr 7-2008. Norwegian Knowledge Centre for the Health Services, Oslo 29320000

[34] Doyle C, Lennox L, Bell D (2013). A systematic review of evidence on the links between patient experience and clinical safety and effectiveness. BMJ Open.

[35] Parand A, Dopson S, Renz A, Vincent C (2014). The role of hospital managers in quality and patient safety: a systematic review. BMJ Open.

[36] Patton M (2015) Purposeful sampling and case selections: overview of strategies and options. Qualitative research and evaluation methods, pp 264–315

[37] Tsianakas V, Robert G, Maben J (2012). Implementing patient-centred cancer care: using experience-based co-design to improve patient experience in breast and lung cancer services. Support Care Cancer.

[38] Doré I, Plante A, Bedrossian N (2022). Developing practice guidelines to integrate physical activity promotion as part of routine cancer care: a knowledge-to-action protocol. PLoS ONE.

[39] Braun V, Clarke V (2006). Using thematic analysis in psychology. Qual Res Psychol.

[40] Birks M, Chapman Y, Francis K (2008). Memoing in qualitative research: probing data and processes. J Res Nurs.

[41] Flottorp SA, Oxman AD, Krause J (2013). A checklist for identifying determinants of practice: a systematic review and synthesis of frameworks and taxonomies of factors that prevent or enable improvements in healthcare professional practice. Implement Sci.

[42] Santa Mina D, Sabiston CM, Au D (2018). Connecting people with cancer to physical activity and exercise programs: a pathway to create accessibility and engagement. Curr Oncol.

[43] Sezgin MG, Bektas H (2022). Effect of peer mentoring on physical activity in patients with cancer: a systematic review and meta-analysis of randomised controlled trials. J Clin Nurs n/a:.

[44] Avancini A, Skroce K, Tregnago D (2020). “Running with cancer”: a qualitative study to evaluate barriers and motivations in running for female oncological patients. PLoS ONE.

[45] Mazzoni A-S, Carlsson M, Berntsen S (2019). “Finding my own motivation” — a mixed methods study of exercise and behaviour change support during oncological treatment. Int J Behav Med.

[46] Mikkelsen MK, Nielsen DL, Vinther A (2019). Attitudes towards physical activity and exercise in older patients with advanced cancer during oncological treatment – a qualitative interview study. Eur J Oncol Nurs.

[47] Sheill G, Guinan E, Neill LO (2018). The views of patients with metastatic prostate cancer towards physical activity: a qualitative exploration. Support Care Cancer.

[48] Smith L, Croker H, Fisher A (2017). Cancer survivors’ attitudes towards and knowledge of physical activity, sources of information, and barriers and facilitators of engagement: a qualitative study. Eur J Cancer Care (Engl).

[49] Chamberlain JM, Haaga DAF (2001). Unconditional self-acceptance and psychological health. J Ration-Emotive Cogn-Behav Ther.

[50] Carlson MA, Fradgley EA, Bridge P (2021). The dynamic relationship between cancer and employment-related financial toxicity: an in-depth qualitative study of 21 Australian cancer survivor experiences and preferences for support. Support Care Cancer.

[51] Blinder VS, Gany FM (2020). Impact of cancer on employment. J Clin Oncol.

